# Below the surface of a touch

**DOI:** 10.7554/eLife.88215

**Published:** 2023-05-12

**Authors:** Stephanie D Preston, Rosa Muñoz

**Affiliations:** 1 Department of Psychology, University of Michigan Ann Arbor United States

**Keywords:** oxytocin, touch, fMRI, Human

## Abstract

How the body and brain respond to a gentle stroke dynamically changes depending on how familiar someone is with the other person.

**Related research article** Handlin L, Novembre G, Lindholm H, Kämpe R, Paul E, Morrison I. 2023. Human endogenous oxytocin and its neural correlates show adaptive responses to social touch based on recent social context. *eLife* **12**:e81197. doi: 10.7554/eLife.81197.

The touch of a partner’s hand slowly stroking across our forearm can be soothing and foster intimacy. Yet the exact same action can feel annoying after an argument, or even creepy when coming from a stranger. How can the brain produce such strong and diverging responses to an identical sensation?

This paradox reflects the fact that our brain evolved as an adaptive and dynamic system that is inherently sensitive to context (Preston, 2022). Specific nerve fibers on the hairy skin of mammals, called C tactile fibers, respond to gentle strokes on the skin that are typically associated with close, bonded relationships. In turn, these cells send signals to brain areas that process sensation, emotion and reward (Löken et al., 2009). The neurohormone oxytocin is known to mediate the pleasure of touch depending on context; it increases in both rodents and humans after a light touch but also interacts with cortisol, the human ‘stress hormone’ which promotes fast metabolic responses to immediate threats (Kurosawa et al., 1995; Li et al., 2019) .

In particular, oxytocin can inhibit this stress response when administered in the laboratory and also when it occurs naturally during mating and caregiving (Heinrichs et al., 2003). For instance, studies in male and female wild chimpanzees revealed that oxytocin increased during inter-group conflicts, with higher levels predicting better cohesion between members of the same community; meanwhile, cortisol, but not oxytocin, also increased with the level of risk or hostility of these intergroup interactions (Samuni et al., 2017; Samuni et al., 2019). Captive marmosets are another example: these animals share more food with opposite-sex strangers than with their paired mate, and display less of this prosocial behavior when administered oxytocin – but only when cortisol levels are already high (Mustoe et al., 2015). This suggests that partner familiarity and context influence how these neurohormones interact across species. However, it has been unclear how these processes play out during typical human social interactions.

Now, in eLife, India Morrison and colleagues – including Linda Handlin and Giovanni Novembre as joint first authors – report new insights into how humans respond to touch in different contexts (Handlin et al., 2023). The team (who are based at Linköping University and the University of Skövde) examined the effects of social touch on oxytocin, cortisol and neural activity. To do so, they placed 42 female participants into a functional neuroimaging scanner where they were alternately touched on the palm or forearm by a partner or a stranger. In parallel, the team measured the levels of oxytocin and cortisol circulating in the participants.

The results confirmed that a partner’s touch was rated as more pleasant, and that it elicited higher oxytocin levels and lower cortisol levels compared to a stranger’s touch. The participants also reported more positive sensations from the arm than the palm, supporting the role of C-fibers in conveying tender touch responses. If the volunteers were first touched by a stranger, the low oxytocin and high cortisol response this event created impeded the typically high oxytocin response to a subsequent partner touch. Conversely, the increase in cortisol that followed a stranger’s touch was lower if a partner had touched the participant first, with oxytocin dipping but recovering quickly to initially high levels. This means that the female participants felt calmer about a typically stressful stranger’s touch if it was preceded by the pleasant touch of a partner but felt less calm to their own partner if a stranger had touched them first. Taken together, these findings suggest that our bodies, brains and emotions respond differently depending on who is touching us, when, and where.

Overall, this sensitivity to context was reflected in the brain activity of the female participants, which also changed in line with self-reported pleasantness and measured hormone levels. For example, activation in the serotonergic dorsal raphe nuclei (which mediates pleasant feelings) and the hypothalamus (which supports social bonding and releases oxytocin) was higher when a partner touched. Participants with higher oxytocin responses also had stronger neural responses to the partner’s touch in the parietotemporal, medial prefrontal and anterior cingulate cortices, which may represent visuo-spatial information associated with the event and the emotion-based response. A region of the medial prefrontal cortex that integrates feelings and decisions was sensitive to changes in both oxytocin and cortisol when the partner touched compared to a stranger.

This greater response to a partner touch before a stranger’s touch was reflected in greater brain activity in the temporal pole and in ratings of pleasantness; activation in this area also increased the more the women rated the second stranger’s touch as pleasant. In an initially less pleasant situation (when the stranger touched first) the dorsal raphe nuclei and hypothalamus were more active when oxytocin levels were low across women, and the amygdala, a brain region that responds during highly salient and fearful events, responded more. In less pleasant situations, such as stranger touching the palm, the brain activity that corresponds to physical sensations and motor responses increased. These data potentially indicate how the unfamiliar touch may have led the participants to increase their focus on that sensation, rather than on their feelings towards it.

Existing research usually examines how brain activity and hormones interact in rodent models (e.g., bonding mates) or in humans engaged in economic games; studies in both species often only test males ( Preston, 2013). Significant work is needed to resolve the many mixed and null findings produced by such experiments, and to segregate how hormones act in the brain versus the body (Churchland and Winkielman, 2012; Heinrichs et al., 2003). Research like the work by Handlin et al., which examines a more natural social situation with multiple concurrent neurophysiological measures, reveals how our brains adaptively and dynamically alter responses based on context. A more nuanced and accurate view of natural social systems can improve models of how bonding shapes how we feel and what we do next. This information could potentially be relevant for people who struggle with social processes.

In a modern world characterized by significant loneliness, frequent interactions with strangers, and touch-free digital socializing, we must better understand the human need for social contact, and our varying responses to those who are close or less close to us. The health and wellbeing of people and society depend upon it.
